# Simian malaria in the Brazilian Atlantic forest: first description of natural infection of capuchin monkeys (Cebinae subfamily) by *Plasmodium simium*

**DOI:** 10.1186/s12936-015-0606-6

**Published:** 2015-02-18

**Authors:** Denise Anete Madureira de Alvarenga, Anielle de Pina-Costa, Taís Nóbrega de Sousa, Alcides Pissinatti, Mariano G Zalis, Martha C Suaréz-Mutis, Ricardo Lourenço-de-Oliveira, Patrícia Brasil, Cláudio Tadeu Daniel-Ribeiro, Cristiana Ferreira Alves de Brito

**Affiliations:** Laboratório de Malária, Centro de Pesquisa René Rachou (CPqRR), Fundação Oswaldo Cruz (Fiocruz), MG Brazil; Instituto Nacional de Infectologia (INI), Ambulatório de Doenças Febris Agudas, Fiocruz, RJ Brazil; Centro de Pesquisa, Diagnóstico e Treinamento em Malária (CPD-Mal), Fiocruz, RJ Brazil; Centro de Primatologia do Rio de Janeiro (CPRJ/INEA), Rio de Janeiro, RJ Brazil; Centro Universitário Serra dos Órgãos (UNIFESO), Rio de Janeiro, RJ Brazil; Laboratório de Virologia Molecular, Hospital Universitário Clementino Fraga Filho, Universidade Federal do Rio de Janeiro, Rio de Janeiro, RJ Brazil; Instituto Oswaldo Cruz, Laboratório de Doenças Parasitárias, Fiocruz, RJ Brazil; Instituto Oswaldo Cruz (IOC), Laboratório de Transmissores de Hematozoários, Fiocruz, RJ Brazil; Laboratório de Pesquisa em Malária (IOC), Fiocruz, RJ Brazil

**Keywords:** Simian malaria, *Plasmodium simium*, *Plasmodium vivax*, *Plasmodium brasilianum*, *Plasmodium malariae*, Cebinae, Molecular diagnosis, Atlantic forest

## Abstract

**Background:**

In Brazil, two species of *Plasmodium* have been described infecting non-human primates, *Plasmodium brasilianum* and *Plasmodium simium*. These species are morphologically, genetically and immunologically indistinguishable from the human *Plasmodium malariae* and *Plasmodium vivax* parasites, respectively. *Plasmodium simium* has been observed naturally infecting monkeys of the genera *Alouatta* and *Brachyteles* in a restricted area of the Atlantic Forest in the south and southeast regions of Brazil. However, its reported geographical distribution and the diversity of its vertebrate hosts may be underestimated, since available data were largely based on analyses by microscopic examination of peripheral blood, a method with limited sensitivity, considering the potential sub-patent feature of these infections. The present study describes, for the first time, the natural infection of *P. simium* in capuchin monkeys from the Brazilian Atlantic Forest.

**Methods:**

Blood samples from 30 non-human primates belonging to nine species kept in the Primate Centre of Rio de Janeiro were collected. Fragments of spleen and liver from one dead monkey found in the neighborhoods of the Primate Centre were also analysed. Molecular diagnosis was performed by nested PCR (18SSU rRNA) and the amplified fragment was sequenced.

**Results:**

Thirty per cent of the captive animals were infected with *P. simium* and/or *P. brasilianum*. The dead monkey tested positive for DNA of *P. simium*. For the first time, Cebinae primates (two specimens of genus *Cebus* and two of genus *Sapajos*) were found naturally infected by *P. simium*. The infection was confirmed by sequencing a small fragment of 18SSU rRNA.

**Conclusion:**

The results highlight the possibility of infection by *P. simium* in other species of non-human primates whose impact could be significant for the malaria epidemiology among non-human primates and, if it becomes clear that this *P. simium* is able to infect monkeys and, eventually, man, also for the maintenance of transmission of human malaria in the context of a zoonosis in areas under influence of the Atlantic Forest.

**Electronic supplementary material:**

The online version of this article (doi:10.1186/s12936-015-0606-6) contains supplementary material, which is available to authorized users.

## Background

Malaria, one of the major public health problems in the world, is a mosquito-borne disease caused by parasites of the genus *Plasmodium* that affects mammals, reptiles and birds [1]. *Plasmodium* species causing infection of non-human primates are of great interest because they may be naturally or accidentally transmitted to humans [2-7]. In Brazil, a country that holds the largest species diversified simian fauna of the planet, only two simian *Plasmodium* species have been found infecting non-human primates: *Plasmodium brasilianum* and *Plasmodium simium*. These species are morphologically, genetically and immunologically indistinguishable from the human malaria parasites *Plasmodium malariae* and *Plasmodium vivax*, respectively [8-11]. Both, *P. brasilianum* and *P. simium* are infective to human [2,12].

*Plasmodium brasilianum* has a wide distribution in Central and South America, where it has been found in Brazil, Colombia, Venezuela, Panama, and Peru naturally infecting a large number of species belonging to all families of New World non-human primates, i.e., Aotidae, Atelidae, Cebidae, and Pitheciidae families [9,13]. In Brazil, excepting the arid portions in the northeast and savannah in the southeast, the territory range of *P. brasilianum* includes all regions and overlaps that of *P. simium,* which is restricted to the Atlantic Forest in the south and southeast [9]. Contrarily to *P. brasilianum*, *P. simium* has been detected infecting only two Atelidae genera: *Alouatta* (howler monkeys) and *Brachyteles* (woolly spider monkeys) [9,14,15]. However, most of the previous simian malaria surveys performed in the Atlantic Forest were based on microscopy of blood smears [9]. Given to the great diversity of primates in the Brazilian Atlantic Forest it is expected that, with the use of more sensitive diagnostic methods, the determination of the real rate of infection in monkeys and, eventually, the expansion of the list of potential hosts would be possible.

According to the National Malaria Control Program (PNCM), Secretary for Health Surveillance (SVS), Ministry of Health, from January 2006 to December 2013, 8,410 cases of malaria were reported in extra-Amazon region, 1,068 of them being autochthonous [16]. In 2013, from the 827 registered extra-Amazonian cases, 10.6% (88 cases) were due to autochthonous transmission, corresponding to 0.05% of the total cases in Brazil [17]. In south and southeastern Brazil, human malaria transmission has been essentially eliminated or has been reported during scattered outbreaks of introduced cases, due to imported ones, for more than four decades [17-19]. However, few autochthonous human malaria cases and outbreaks have been reported associated with the Atlantic Forest environment, particularly in its mountain valleys in southeast Brazil [9,17,19,20]. In such environments, malaria transmission is supported by the bromeliad mosquito *Anopheles (Kerteszia) cruzii,* which has been proved to be the natural vector of both human and simian malaria in south and southeast Brazil [9]. Therefore, Deane and colleagues in the 1990s and others [8,9,15,17,21] have suggested that human malaria in the Atlantic Forest could be a zoonosis where non-human primates are the reservoirs.

Autochthonous human malaria cases reported in the mountain valleys of the Atlantic Forest environment in Rio de Janeiro State motivated the search for natural simian plasmodial infections in this region [17]. The present study describes for the first time the natural infection of *P. simium* in the subfamily Cebinae by using molecular approaches.

## Methods

### Origin of primate specimens

**Figure 1:**
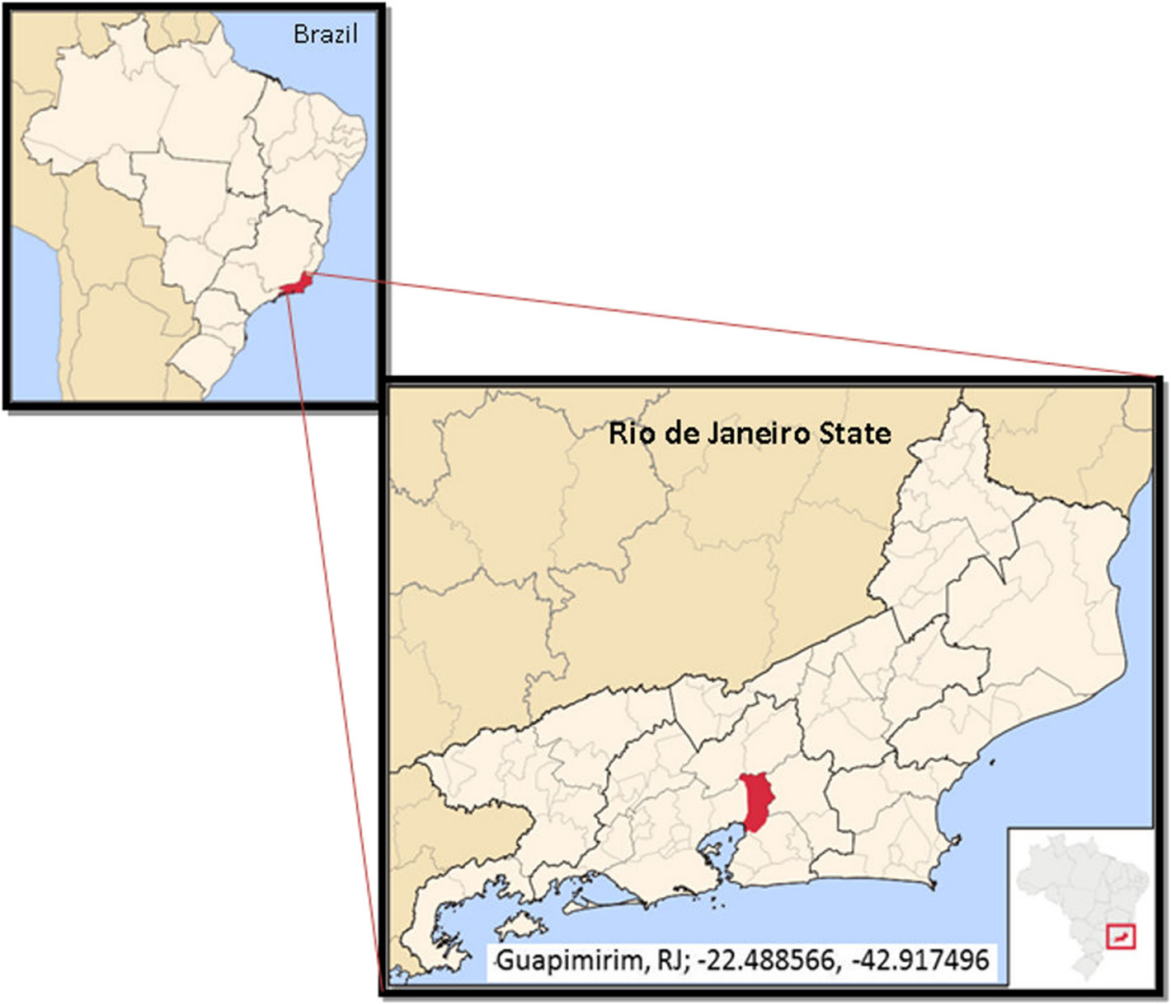
**Maps of Brazil and of the State of Rio de Janeiro.** Municipality of Guapimirim and its coordinates (red in Rio de Janeiro State map), in Serra dos Órgãos, where the Primate Centre of Rio de Janeiro (CPRJ) is located, in an area under influence of the Atlantic Forest, about l00 km from the city of Rio de Janeiro. Figure modified from Wikimedia Commons: http://commons.wikimedia.org/wiki/File:RiodeJaneiro_Municip_Guapimirim.svg.

Animals used in this study were captive primates from the Primate Centre of Rio de Janeiro (CPRJ). The CPRJ (IBAMA register number 458460) is a unit for wild monkey protection and is located in the municipality of Guapimirim, on the Serra dos Órgãos slopes (Figure 1), in an area under influence of the Atlantic Forest, about l00 km from the city of Rio de Janeiro. Serra dos Órgãos is part of the large coastal mountain chain in southeast Brazil named Serra do Mar. One wild animal, identified as an adult female of *Alouatta guariba clamitans*, was found dead nearby the CPRJ in December 2013 and an autopsy was performed, and spleen and liver fragments were included in the analysis. The present study was conduced in response to a demand of the Health Secretary of Rio de Janeiro State, to the Centro de Pesquisa, Diagnóstico e Treinamento em Malária (CPD-Mal), Fiocruz, in the context of an investigation on the autochthonous human cases registered in the surroundings of the CPRJ, and it had, therefore, the authorization of the Fiocruz Human Ethical Committee. As the handling of monkeys was exclusively done by CPRJ technicians, Fiocruz Animal Ethics Committee (CEUA) agreed to the protocol for sample collection.

### Samples collection and DNA extraction

A total of 30 captive primates were sampled (Additional file 1) in November 2011. Of these, 18 were female (62%); the average age was six years, median of seven years (1–14). The average weight was of 2,800 g (700–7,500 g). No animal was splenectomized at the time of sample collection. Approximately 2 ml of blood were taken in vacuum tubes containing EDTA by femoral venipuncture. For just a few individuals sedation with Quetamina® and Midazolan was required. The samples collection was performed by the Med Vet Alcides Pissinatti, the blood samples were frozen and send to the Malaria laboratory of the CPqRR, Fiocruz.

DNA extraction from blood samples was made using the Gentra Puregene Blood kit (Qiagen, Venlo, The Netherlands) according to manufacturer’s recommendations. Fragments of spleen and liver from the dead monkey were used for DNA extraction using the Gentra Puregene kit (Qiagen, Venlo, The Netherlands) according to manufacturer’s recommendations. The DNA was eluted in a 100 μL volume and stored at −20°C until it was used.

### Nested-PCR

The samples were subjected to nested-PCR [22] using primers for identification of human *Plasmodium* species, targeting the small subunit of 18S ribosomal RNA gene (18SSU rRNA). Briefly, all PCR reactions were performed in 20 μL volumes containing 250 μM each oligonucleotide primer, 10 μL of Master Mix (Promega) (0.3 units of Taq Polymerase, 200 μM each deoxyribonucleotide triphosphates and 1.5 mM MgCl_2_) and 2 μL DNA. The PCR assays were performed using an automatic thermocycler (PTC-100TM v.7.0) (MJ Research Inc, USA) and the following cycling parameters were used: an initial denaturation at 95°C for 5 min followed by 24 cycles of annealing at 58°C for 2 min, extension at 72°C for 2 min and denaturation at 94°C for 1 min followed by a final annealing incubation at 58°C for 2 min and extension at 72°C for 2 min. The temperature was then reduced to 4°C until the samples were taken. The cycling parameters for the second round of PCR were the same as the first reaction, but instead 30 cycles of amplification were used.

To prevent cross-contamination, the DNA extraction and master mix preparation were performed in “parasite DNA-free rooms” distinct from each other. Furthermore, each of these separate areas has different sets of pipettes and all procedures were performed using plugged pipette tips. DNA extraction was performed twice at different days. Every PCR reaction had a negative control, where DNA has replaced by water and also positive controls for each pair of primers. The sources of genomic DNA samples that served as positive controls in the nested PCR assays are: i) *P. falciparum* DNA*,* strain 3D7 maintained in Malaria Laboratory (CPqRR-FIOCRUZ MINAS); (ii) DNA of patient with high parasitaemia for *P. vivax* and DNA of *P. simium* of a non-human primate with an acute infection and parasitaemia confirmed by optical microscopy (BL10); (iii) DNA of *P. brasilianum* of MR4 (Malaria Research and Reference Reagent Resource Center – ATCC, USA).

The amplified fragments were visualized in electrophoresis on 1.5% agarose gel dissolved in 1x TAE buffer (40 mM Tris-acetate, 1 mM EDTA) with 5 μg/mL ethidium bromide (Invitrogen) in a horizontal system (Bio-Rad) at 100 V for about 30 min. The gels were be examined under UV transilluminator (UVP - Bio-Doc System it) and were filed under digital system. Electrophoresis was performed in a room specific for amplified DNA, with appropriated sets of pipettes and plugged pipettes tips.

### Sequencing of PCR-amplified DNA

For DNA sequencing, PCR products were purified using Purification Kit QIAquick (Qiagen) following manufacturer’s procedure. Around 3 ng of purified PCR products were amplified using 2.0 μM of each primer (forward or reverse of species-specific primers of second reaction) and 1 μL of Big Dye terminator kit in a program of: 96°C for 1 min, 35 cycles of 96°C for 15 sec, the temperature of primer annealing for 15 sec and 60°C for 15 sec. The fragments were precipitated using ammonium acetate, ressuspended in formamide HI-DI (Applied Biosystems) and electrophoretically separated in ABI 3730 DNA automatic sequencer.

### Data analysis

The sequence data were analysed using the Blast program and the sequences aligned using the Clustal W and CAP3 programs in Bioedit package and also Muscle multiple alignment program [23]. The tree was constructed using the maximum likelihood method with Tamura 3-parameter model [24] and 5,000 bootstrap replicates in MEGA 6.0 software [25].

## Results

**Figure 2:**
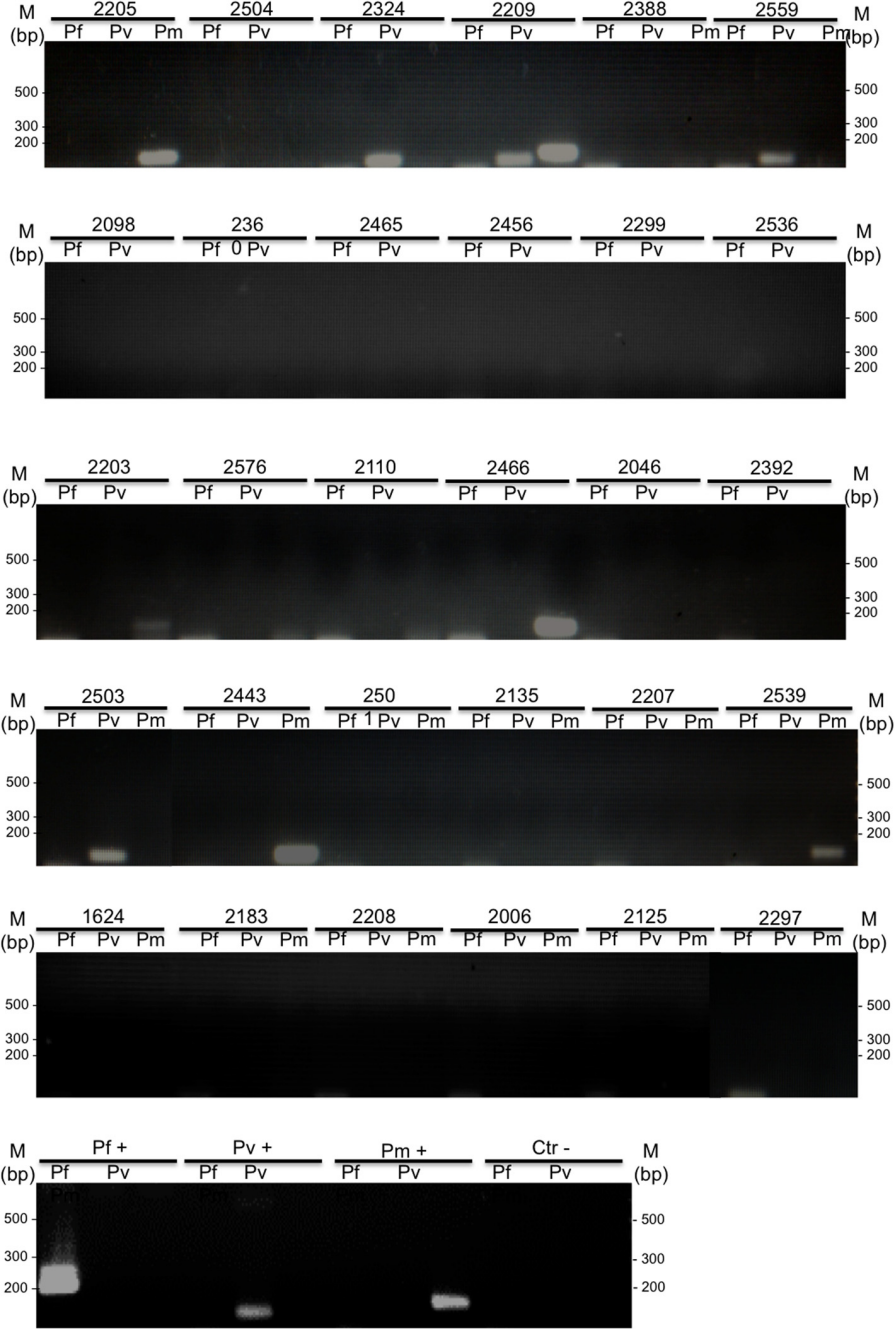
**Molecular diagnosis of** ***Plasmodium*** **infection by nested-PCR of 30 non-human primate samples.** Molecular diagnosis of *Plasmodium* infection by nested-PCR in 30 non-human primates from CPRJ using specific primers for *P. falciparum* (Pf), *P. vivax* (Pv) and *Plasmodium malariae* (Pm). *Plasmodium simium* was identified in *Sapajus xanthosternos* (2324), *Sapajus robustus* (2209) and *Cebus* sp. (2503 and 2559); and *P. brasilianum* was detected in *Sapajus xanthosternos* (2005 and 2539), *Callicebus personatus* (2466)*, Aotus nigriceps* (2203), *Sapajus robustus* (2209) and *Alouatta g. clamitans* (2443). Agarose gels, 1.5% stained with ethidium bromide. Numbers above gels indicated the non-human primates according to Additional file 1. Pf+, Pv + and Pm+: positive control (DNA species-specific) and Ctr -: negative control (without DNA), MM – 1 kb Plus Ladder.

**Figure 3:**
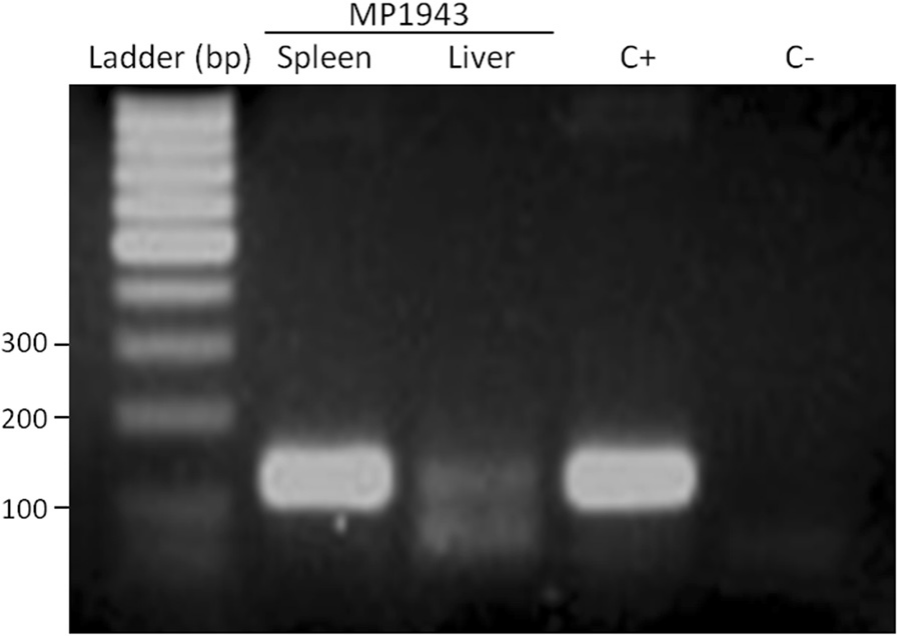
**Molecular diagnosis of** ***Plasmodium*** **infection by nested-PCR in organ fragments of dead monkey.** DNA extracted from spleen and liver samples of dead wild *Alouatta g. clamitans* found near the campus of the CPRJ (sample MP1943) was amplified by using primers specific for *P. vivax* (Pv). Agarose gel, 1.5% stained with ethidium bromide. C+ − positive control for *P. vivax* and CN – negative control (without DNA), M – Ladder 100 bp.

Molecular diagnosis was performed in samples from 30 non-human primates from the CPRJ, and nine samples (30%) were found to be positive by nested-PCR: five samples were positive for *P. brasilianum*; three samples were positive for *P. simium* and one sample was positive for both *P. simium* and *P. brasilianum* (Figure 2). Negative controls never showed any amplified fragments and positive controls showed fragments with the expected sizes (Figure 2). *Plasmodium simium* was identified in *Sapajus xanthosternos*, *Sapajus robustus* and *Cebus* sp.; and *P. brasilianum* was detected in *Sapajus xanthosternos*, *Sapajus robustus*, *Callicebus personatus, Aotus nigriceps* and *Alouatta g. clamitans* (see Additional file 1). The dead wild monkey found nearby the CPRJ, sample MP1943, an *Alouatta g. clamitans,* was also found positive for *P. simium* by using DNA obtained from the spleen fragment and, with less intensity of amplification, from the liver fragment (Figure 3).

**Figure 4:**
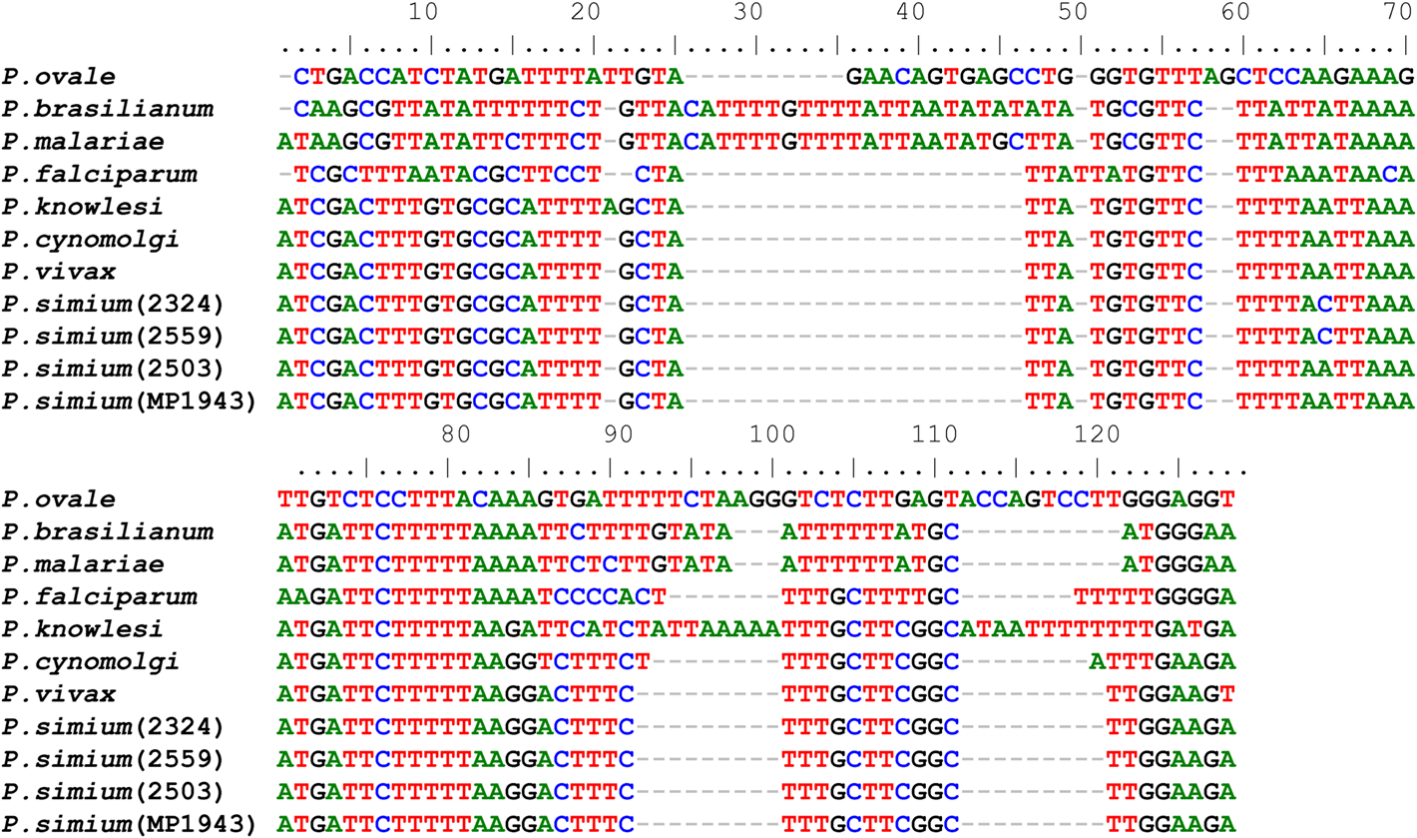
**Multiple sequence alignment of** ***Plasmodium*** **18SSU rRNA fragment performed by the muscle algorithm.** The 18SSU rRNA partial sequences (second amplicon from Nested-PCR) obtained herein are from parasites of: *Sapajus xanthosternos* (sample 2324), *Cebus *sp. (2559 and 2503), dead wild *Alouatta g. clamitans* (MP1943). These sequences were compared to sequences from *Plasmodium* species available in GenBank [accession number]: *P. vivax* [GenBank: AY579418.1], *Plasmodium cynomolgi* [Genbank:JQ794445.1], *Plasmodium knowlesi* [GenBank: AY579417], *P. falciparum* [GenBank: JQ627150.1], *P. malariae* [GenBank: GU815531.1], *P. brasilianum* [GenBank: KC906730.1] and *Plasmodium ovale* [GenBank: KF018663.1].

**Figure 5:**
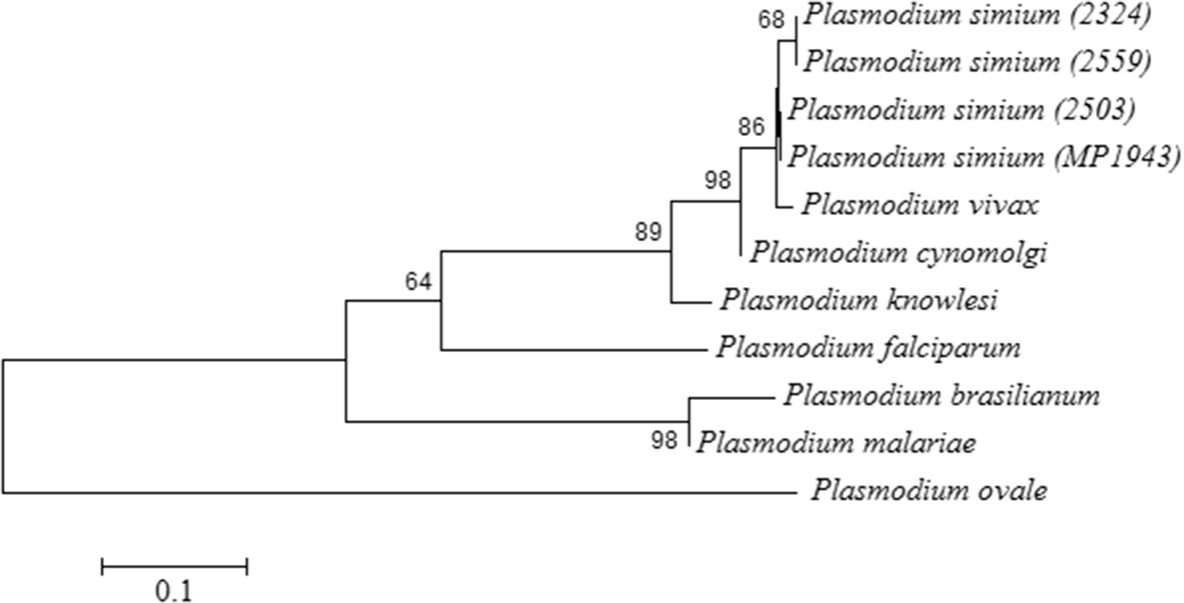
**Topology of the phylogenetic tree of 18SSU rRNA**. Topology of the phylogenetic tree of 18SSU rRNA with the four sequences of *P. simium* obtained from*: Sapajus xanthosternos* (2324), *Cebus *sp. (2503 and 2559) and death wild *Alouatta g. clamitans* (MP1943). The same sequences from the other species were used as in Figure 4. The tree was generated using the method of maximum likelihood and Tamura 3-parameter model with 5,000 replicates (bootstraps >60% showed in the branches).

A species-specific fragment of 18SSU rRNA from *P. simium* identified in the molecular diagnosis was sequenced to confirm the infection for *P. simium* in monkeys from the Cebinae subfamily. Then the nucleotide sequence of 18SSU rRNA fragments from four infected animals (2324, 2559, 2503, and MP1943) identified here were compared to *P. vivax* and other *Plasmodium* species sequences available at Genbank (Figure 4). From the alignment, the high genetic similarity between *P. simium* and *P. vivax* was evident (one difference out of 86 nt). It was also able to discern between the *P. vivax/P. simium* from the other species of *Plasmodium*. In addition, this alignment was used to reconstruct the phylogenetic relationships of the simian and human *Plasmodium*. *Plasmodium simium* sequences clustered in a single branch. Moreover, all samples of *P. simium* are in the clade of *P. vivax*, reinforcing the genetic similarity between these parasites (Figure 5).

## Discussion

By molecular analysis of blood samples of 30 captive primates from a primate center in southeastern Brazil, 30% of the individuals were found infected with *Plasmodium.* Moreover, the dead monkey (*Alouatta clamitans*) found close to the CPRJ and also studied here showed infection by *P. simium* through detection of *Plasmodium* DNA in spleen and liver fragments. Interestingly, previous surveys based on microscopy of simian blood smears conducted in the same primate center (CPRJ) as well in other localities of the state of Rio de Janeiro failed to detect *Plasmodium* infections [26,27].

Although microscopic slides were not obtained, the presence of parasite DNA, even indicating possibly a low parasitaemia in the examined animals, points to the identification of a new natural host of *P. simium*: the capuchins monkeys. Capuchins monkeys are New World monkeys of the family Cebidae, subfamily Cebinae, that have the largest geographical distribution among the neotropical primates [28-32]. Prior to 2011, the subfamily Cebinae contained only a single genus, *Cebus*. However, recent studies have suggested the division of capuchin monkeys into two genera, *Sapaju*s for robust, tufted capuchins and *Cebus* for untufted, gracile capuchins [33]. *Cebus* and *Sapajus* have been described infected only by *P. brasilianum* [9,34]. However, this study describes that primates belonging to three species from both genera were found infected by *P. simium*. According to the literature, *P. simium* was described infecting only two species of genus *Alouatta* and one of *Brachyteles* primates [9,15]. Also, no evidence of experimental infection was obtained in splenectomized *Cebus* sp. inoculated with *P. simium* heavily parasitized blood sample [35]. Based on microscopy of blood smears, the natural infection rates for *P. brasilianum* in capuchin monkeys are usually very low compared to other Cebidae species, such as squirrel monkeys, as well as Pitheciidae and Atelidae species [9,13]. Thus, the finding of *P. simium* DNA in blood samples of capuchin monkeys from Rio de Janeiro suggests that these primates possibly undergoes *Plasmodium* infections with very low or transient parasitaemia.

Although the prevalence of Cebidae monkeys found infected in the CPRJ, which is located inside the Atlantic Forest, is similar to those reported for other species of monkeys found to carry *Plasmodium* parasites in the region [9,15,21], it is still necessary to determine if captive monkeys were caught infected by *Plasmodium* or if the transmission has occurred at the CPRJ, facilitated by the simultaneous presence of the vector and infected monkeys. *Plasmodium* infection detected in one monkey born in CPRJ (monkey 2324), the naturally infected (dead) wild monkey in the neighborhoods of CPRJ and the existence of malaria vectors such as *An. (Ker.) cruzii*, *Anopheles (Nyssorhynchus) albitarsis* and *Anopheles (Nyssorhynchus) aquasalis* in the region may support the latter hypothesis [36,37]. In addition, the high proportion of monkeys found carrying antibodies against blood forms and sporozoites of *P. brasilianum* and *P. simium* may either reinforce the hypothesis of an increased transmission in the CPRJ neighborhoods or suggest that the prevalence of *Plasmodium* infection may be much higher than expected in this area of Atlantic Forest [9,15,21,38].

As all measures to avoid contamination have been undertaken here and the PCR reactions were very well controlled (see Methods) the results presented here can not result from cross-contamination in the laboratory. Therefore, the present list of *P. simium*-susceptible, neotropical, non-human primate species might be incomplete because of the usual nature of sub-patent infections along with the limited sensitivity of the diagnostic methods used, that were mainly based on analyses of peripheral blood microscopy. The findings showed here are strengthened by those of Duarte *et al.* who described the occurrence of antibodies against the *P. vivax* circumsporozoite surface protein (CSP) in *Cebus* sp. from Brazil [11]. The presence of individuals from the *Cebus* genus carrrying antibodies against *P. vivax* DBP_II_, MSP1_19_ and AMA1 has also been identified by our group in monkeys from different areas (unpublished data).

New World simian *Plasmodium* are very closely related to human *Plasmodium*; *P. brasilianum* and *P. simium* are similar to the human *P. malariae* and *P. vivax* parasites, respectively. The sequencing of a small fragment of the18SSU rRNA encoding gene showed a high genetic similarity between *P. vivax* and *P. simium*, as demonstrated by Tazi and Ayala [39]. This high identity is also observed in the phylogenetic reconstruction of *P. simium* isolates studied here. It is important to notice that the small size of the sequenced fragment may hinder their ability to reproduce the phylogeny of the genus *Plasmodium*. Moreover, the high level of conservation in 18SSU rRNA among close species, such as *P. vivax* and *P. simium,* hamper the discussion if these species are distinct or not from each other. Many authors based on an analysis of different molecular targets, such as the conserved regions of the gene coding for the circumsporozoite surface protein (CSP) or mitochondrial genes have been suggesting the existence of two different species, *P. simium* and *P. vivax*, very closely related and their origin was through multiple host switches recently in the evolution [40-42].

The evolutionary history of *P. vivax* in the Americas is still very controversial. The most accepted hypothesis is that of post-Columbian origin, where *P. vivax* would have arrived with Europeans during the colonization of the Americas [43,44]. In the New World, several evidences suggest a recent introduction of *P. vivax* [43,45,46]. The evolutionary history of *P. vivax* in the Americas was studied by analysing the mitochondrial genome variability of these parasites. Many different genetic groups circulating in South America were found, and they have probably originated from independent introductions [47].

Despite the data presented here is not sufficient to support a deep discussion about this subject, the evidence collected so far suggest that *P. simium* is possibly a different species from *P. vivax*, based especially on some differences on the morphology and, more recently, polymorphisms in some genomic sequences. However, additional genomic information on *P. simium* is still needed to support this hypothesis.

Taken together, the data reinforce the hypothesis that non-human primates of Atlantic Forest, including species which have never be incriminated before, such as the capuchins monkeys, could be acting as a reservoir for *Plasmodium*. Once autochthonous human cases of malaria are reported in those regions, the presence of possible wild reservoirs may have important implications for public health, due to close contact between humans and monkeys in some parts of the Atlantic Forest environment [17,19].

## Conclusions

The findings presented here might impact the knowledge on the epidemiology of the disease among human and non-human primates in the Brazilian Atlantic Forest. If it becomes clear that the same parasite can infect both humans and monkeys in this area, the presence of possible animal reservoirs may have important implications for public health [48] and areas of Atlantic Forest should be under strict surveillance for the possibility of a zoonotic pattern of malaria transmission. However, more studies are still needed to confirm this, particularly the detection of gametocytes in the monkeys and the identification of the same parasites circulating among the vectors and both hosts: human and simian. Due to the known close similarity between *P. simium* and *P. vivax*, the data reported here indicate that *Cebus* and *Sapajus* monkeys should have their susceptibility to *P. vivax* investigated in order to determine if they could represent new experimental models for studies with human *Plasmodium*, in addition to the *Saimiri* and *Aotus* neotropical monkeys recommended by the World Health Organization [49].

## Additional file

Additional file 1:
**Description of 30 non-human primates studied and molecular diagnosis of** ***Plasmodium*** **.**

## References

[1] Perkins SL, Austin CC (2009). Four new species of *Plasmodium* from New Guinea lizards: integrating morphology and molecules. J Parasitol.

[2] Deane LM, Deane MP, Ferreira Neto J (1966). Studies on transmission of simian malaria and on the natural infection of man with *Plasmodium simium* in Brazil. Bull World Health Organ.

[3] Arruda ME, Nardini EH, Nussenzweig RS, Cochrane AH (1989). Sero-epidemiological studies of malaria in indian tribes and monkeys of the Amazon basin of Brazil. Am J Trop Med Hyg.

[4] Carréri-Bruno GC, Ciaravolo RM, Pereira M (1995). Malaria acquired during entomological research in the Serra do Mar, southeastern region of Brazil. Rev Saude Publica.

[5] Singh B, Kim Sung L, Matusop A, Radhakrishnan A, Shamsul SS, Cox-Singh J (2004). A large focus of naturally acquired *Plasmodium knowlesi* infections in human beings. Lancet.

[6] Ta TH, Hisam S, Lanza M, Jiram AI, Ismail N, Rubio JM (2014). First case of a naturally acquired human infection with *Plasmodium cynomolgi*. Malar J.

[7] Yusof R, Lau YL, Mahmud R, Fong MY, Jelip J, Ngian HU (2014). High proportion of knowlesi malaria in recent malaria cases in Malaysia. Malar J.

[8] Coatney GR (1971). The simian malarias: zoonoses, anthroponoses, or both?. Am J Trop Med Hyg.

[9] Deane LM (1992). Simian malaria in Brazil. Mem Inst Oswaldo Cruz.

[10] Leclerc M, Hugot J, Durand P, Renaud F (2004). Evolutionary relationships between 15 *Plasmodium* species from new and old world primates (including humans): an 18S rDNA cladistic analysis. Parasitology.

[11] Duarte AM, Porto MA, Curado I, Malafronte RS, Hoffmann EH, de Oliveira SG (2006). Widespread occurrence of antibodies against circumsporozoite protein and against blood forms of *Plasmodium vivax*, *P. falciparum* and *P. malariae* in Brazilian wild monkeys. J Med Primatol.

[12] Garnham PCC (1966). Malaria parasites and other haemosporidia.

[13] Lourenço-de-Oliveira R, Deane LM (1995). Simian malaria at two sites in the Brazilian Amazon. I – The infection rates of Plasmodium brasilianum in non-human primates. Mem Inst Oswaldo Cruz.

[14] Deane LM, Ferreira Neto J, Sitônio JG (1968). A new natural host of *Plasmodium simium* and *Plasmodium brasilianum*: the woolly spider monkey. Rev Inst Med Trop Sao Paulo.

[15] Duarte AM, Malafronte RS, Cerutti C, Curado I, de Paiva BR, Maeda AY (2008). Natural *Plasmodium* infections in Brazilian wild monkeys: reservoirs for human infections?. Acta Trop.

[16] SIVEP [www.saude.gov.br/svs].

[17] Pina-Costa A, Brasil P, Santi SM, Araujo MP, Suárez-Mutis MC, Santelli AC (2014). Malaria in Brazil: what happens outside the Amazonian endemic region. Mem Inst Oswaldo Cruz.

[18] Deane LM (1988). Malaria studies and control in Brazil. Am J Trop Med Hyg.

[19] Oliveira-Ferreira J, Lacerda MV, Brasil P, Ladislau JL, Tauil PL, Daniel-Ribeiro CT (2010). Malaria in Brazil: an overview. Malar J.

[20] Miguel RB, Peiter PC, Albuquerque H, Coura JR, Moza PG, Costa Ade P (2014). Malaria in the state of Rio de Janeiro, Brazil, an Atlantic Forest area: an assessment using the health surveillance service. Mem Inst Oswaldo Cruz.

[21] Yamasaki T, Summa ME, Neves DV, de Oliveira SG, Gomes AC (2008). Natural *Plasmodium* infections in Brazilian wild monkeys: reservoirs for human infections?. Acta Trop.

[22] Snounou G, Viriyakosol S, Jarra W, Thaithong S, Brown KN (1993). Identification of the four human malaria parasite species in field samples by the polymerase chain reaction and detection of a high prevalence of mixed infections. Mol Biochem Parasitol.

[23] Bioedit (http://www.mbio.ncsu.edu/bioedit/bioedit.html).

[24] Tamura K (1992). Estimation of the number of nucleotide substitutions when there are strong transition-transversion and G + C-content biases. Mol Biol Evol.

[25] Tamura K, Peterson D, Peterson N, Stecher G, Nei M, Kumar S (2011). MEGA5: molecular evolutionary genetics analysis using maximum likelihood, evolutionary distance, and maximum parsimony methods. Mol Biol Evol.

[26] Lourenço-de-Oliveira R (1990). Natural infection of golden lion tamarin, *Leontopithecus rosalia*, with *Trypanosoma cruzi* in the state of Rio de Janeiro. Mem Inst Oswaldo Cruz.

[27] Moreira GV, Peixoto CMS, Ziccardi M, Oliveira RL, Castro MG, Dionísio DF (2000). Prevalência de *Trypanosoma cruzi, Trypanosoma minasense* e de anticorpos contra arbovírus em primatas não humanos (Callithrichidae) em cativeiro. Rev Bras Med Vet.

[28] Fragaszy DM, Visalberghi E, Fedigan LM (2004). The complete Capuchin – the biology of the genus Cebus.

[29] Izawa K (1979). Foods and feeding behavior of wild black-capped capuchin (*Cebus apella*). Primates.

[30] Freese CHE, Oppenheimer JR, A. & R. Mittermeier (1981). The Capuchin Monkeys, Genus *Cebus*. Ecology and behavior of neotropical primates. Volume 1.

[31] Brown A, Chalukian S, Malmierca L (1984). Habitat y alimentacion de *Cebus apella* en el N.O. Argentino y la disponibilidad de frutos en el dosel arboreo. Revista del Museuo Argentino de Ciencias Naturales.

[32] Fedigan LM (1990). Vertebrate predation in *Cebus capucinus*: meat eating in a Neotropical monkey. Folia Primatol.

[33] Alfaro JW, Silva JD, Rylands AB (2012). How different are robust and gracile capuchin monkeys? An argument for the use of *Sapajus* and *Cebus*. Am J Primatol.

[34] Araújo MS, Messias MR, Figueiró MR, Gil LH, Probst CM, Vidal NM (2013). Natural Plasmodium infection in monkeys in the state of Rondônia (Brazilian Western Amazon). Malar J.

[35] Deane LM (1964). Studies on simian malaria in Brazil. Bull World Health Organ.

[36] Flores-Mendoza C, Lourenço-de-Oliveira R (1996). Bionomics of *Anopheles aquasalis* Curry 1932, in Guaraí, State of Rio de Janeiro, southeastern Brazil-I. Seasonal distribution and parity rates. Mem Inst Oswaldo Cruz.

[37] Miguel RB (2011). Estudo da infecção humana por *Plasmodium* spp no município de Guapimirim, estado do Rio de Janeiro.

[38] Curado I, Dos Santos MR, de Castro Duarte AM, Kirchgatter K, Branquinho MS, Bianchi Galati EA (2006). Malaria epidemiology in low-endemicity areas of the Atlantic Forest in the Vale do Ribeira, São Paulo, Brazil. Acta Trop.

[39] Tazi L, Ayala FJ (2011). Unresolved direction of host transfer of *Plasmodium vivax* v. *P. simium* and *P. malariae* v. *P. brasilianum*. Infect Genet Evol.

[40] Escalante AA, Barrio E, Ayala FJ (1995). Evolutionary origin of human and primate malarias: evidence from the circumsporozoite protein gene. Mol Biol Evol.

[41] Escalante AA, Cornejo OE, Freeland DE, Poe AC, Durrego E, Collins WE (2005). A monkey’s tale: the origin of *Plasmodium vivax* as a human malaria parasite. Proc Natl Acad Sci U S A.

[42] Mu J, Joy DA, Duan J, Huang Y, Carlton J, Walker J (2005). Host switch leads to emergence of *Plasmodium vivax* malaria in humans. Mol Biol Evol.

[43] Carter R (2003). Speculations on the origins of *Plasmodium vivax* malaria. Trends Parasitol.

[44] Lim C, Tazi L, Ayala F (2005). *Plasmodium vivax*: recent world expansion and genetic identity to *Plasmodium simium*. Proc Natl Acad Sci U S A.

[45] Cornejo OE, Escalante AA (2006). The origin and age of *Plasmodium vivax*. Trends Parasitol.

[46] Li J, Collins WE, Wirtz RA, Rathore D, Lal A, McCutchan TF (2001). Geographic subdivision of the range of the malaria parasite *Plasmodium vivax*. Emerg Infect Dis.

[47] Taylor JE, Pacheco MA, Bacon DJ, Beg MA, Machado RL, Fairhurst RM (2013). The evolutionary history of *Plasmodium vivax* as inferred from mitochondrial genomes: parasite genetic diversity in the Americas. Mol Biol Evol.

[48] Pacheco MA, Cranfield M, Cameron K, Escalante AA (2013). Malarial parasite diversity in chimpanzees: the value of comparative approaches to ascertain the evolution of *Plasmodium falciparum* antigens. Malar J.

[49] World Health Organization (WHO) (2004). MALVAC meeting 2004: evaluation of malaria vaccines. Pre-clinical evaluation group: optimizing the developmental pathway from the lab to the clinic.

