# Supplementary material for: Simian malaria in the Brazilian Atlantic forest: first description of natural infection of capuchin monkeys (Cebinae subfamily) by Plasmodium simium
Source: Malar J. 2015 Feb 18;14:81. doi: 10.1186/s12936-015-0606-6 (PMC4342080; doi:10.1186/s12936-015-0606-6)
Supplement: Additional file 1: — Description of 30 non-human primates studied and molecular diagnosis of Plasmodium . [file 12936_2015_606_MOESM1_ESM.docx]

**Table S1 Description of 30 non-human primates studied and molecular diagnosis of *Plasmodium***

| **Code** | **Species** | **Sex** | **Weight (kg)** | **Age (years)** | **Origin** | **Date of arriving or birth in CPRJ** | **Molecular Diagnosis** |
| --- | --- | --- | --- | --- | --- | --- | --- |
| **MP1943** | ***Alouatta guariba clamitans*** | **F** | **3.7** | **10.0** | **Wild, found dead nearby CPRJ** | 04 /12 /2013 | ***P. simium*** |
| 1624 | *Sapajus xanthosternos* | M | 5.2 | 16.0 | Born in CPRJ | 30/11/1995 | Negative |
| **2005** | ***Sapajus xanthosternos*** | **F** | **2.9** | **13.0** | **Wild captured in Ilhéus, BA** | **15/12/2000** | ***P. brasilianum*** |
| 2006 | *Sapajus xanthosternos* | F | 3.0 | 13.0 | Ilhéus, BA | 15/12/2000 | Negative |
| 2046 | *Sapajus xanthosternos* | F | 2.6 | 9.5 | Born in CPRJ | 07/01/2002 | Negative |
| 2098 | *Sapajus xanthosternos* | F | 2.4 | 9.0 | Born in CPRJ | 25/11/2002 | Negative |
| 2110 | *Sapajus robustus* | F | 3.2 | 8.5 | Captured in Belo Horizonte, MG | 07/04/2003 | Negative |
| 2125 | *Sapajus xanthosternos* | F | 3.1 | 8.0 | Born in CPRJ | 06/10/2003 | Negative |
| 2135 | *Sapajus xanthosternos* | F | 2.3 | 7.6 | Born in CPRJ | 15/02/2004 | Negative |
| 2183 | *Sapajus xanthosternos* | F | 2.0 | 6.1 | Born in CPRJ | 20/12/2004 | Negative |
| **2203** | ***Aotus nigriceps*** | **M** | **0.7** | **7.0** | **Breeding site in Velho Jatobá, Sorocaba, SP** | **11/04/2005** | ***P. brasilianum*** |
| 2207 | *Sapajus robustus* | M | 3.5 | 9.0 | Wild captured in Porto Seguro, BA | 05/05/2005 | Negative |
| 2208 | *Sapajus robustus* | F | 2.9 | 9.0 | Wild captured in Porto Seguro, BA | 05/05/2005 | Negative |
| **2209** | ***Sapajus robustus*** | **M** | **2.2** | **7.0** | Wild captured in Porto Seguro, BA | 05/05/2005 | ***P. simium and P. brasilianum*** |
| 2297 | *Sapajus robustus* | F | 2.0 | 8 | Born in CPJR | 21/04/2003 | Negative |
| 2299 | *Saimiri sciureus* | M | 0.8 | 6.0 | Cachoeiras de Macacu, RJ, impounded from illegal captivity | 03/07/2007 | Negative |
| **2324** | ***Sapajus xanthosternos*** | **F** | **2.45** | **3.5** | Born in CPRJ | **10/01/2008** | ***P. simium*** |
| 2360 | *Sapajus robustus* | F | 2.2 | 3.0 | Born in CPRJ | 06/10/2008 | Negative |
| 2388 | *Sapajus xanthosternos* | M | 2.4 | 3.0 | Born in CPRJ | 22/11/2008 | Negative |
| 2392 | *Sapajus xanthosternos* | F | 1.2 | 2.1 | Born in CPRJ | 08/12/2008 | Negative |
| **2443** | ***Alouatta fusca clamitans*** | **M** | **6.25** | **5.0** | **Wild captured in Cachoeira de Macacu, RJ** | **28/09/2009** | ***P. brasilianum*** |
| 2456 | *Sapajus robustus* | M | 1.8 | 2.0 | Born in CPJR | 18/12/2009 | Negative |
| 2465 | *Callicebus moloch donacophilus* | F | 1.6 | 3.0 | Zoo Ecological park of Quinzinho de Barros, SP | 25/01/2009 | Negative |
| **2466** | **Callicebus personatus** | **F** | **1.0** | **4.0** | **Wild catched in Belo Horizonte, MG, transferred to Zoo of Sorocaba, SP** | **25/01/2010** | ***P. brasilianum*** |
| 2501 | *Cebus sp.* | M* | 3.1 | 2.5 | Parque dos Passaros, Rio das Ostras, RJ | 09/06/2010 | Negative |
| **2503** | ***Cebus sp.*** | **F*** | **2.4** | **15** | **Triage center of wild animals, Viçosa, MG, wild parents** | **22/07/2010** | ***P. simium*** |
| 2504 | *Cebus sp.* | F* | 2.1 | 2.5 | Triage center of wild animals, Viçosa- MG | 22/07/2010 | Negative |
| **2559** | ***Cebus sp.*** | **F*** | **2.3** | **5.5** | **Zoologico of Bom Jardim, RJ** | **15/06/2011** | ***P. simium*** |
| 2536 | *Sapajus xanthosternos* | M | 1.7 | 1.0 | Born in CPRJ | 19/10/2010 | Negative |
| **2539** | ***Sapajus xanthosternos*** | **M** | **2.3** | **4.0** | **Born in CPRJ** | **ND** | ***P. brasilianum*** |
| 2576 | *Alouatta caraya* | M | 7.5 | 4.0 | Recreio dos Bandeirantes, Estácio de Sá, RJ | 11/11/2011 | Negative |

*Hybrids, ND – not determined
